# Long range correlations of the ion current in SV channels. Met_3_PbCl influence study

**DOI:** 10.1371/journal.pone.0229433

**Published:** 2020-03-03

**Authors:** Janusz Miśkiewicz, Zenon Trela, Zbigniew Burdach, Waldemar Karcz, Wanda Balińska-Miśkiewicz

**Affiliations:** 1 Institute of Theoretical Physics, University of Wrocław, Wrocław, Poland; 2 Department of Physics and Biophysics, Wrocław University of Environmental and Life Sciences, Wrocław, Poland; 3 Department of Plant Physiology, Faculty of Biology and Environmental Protection, University of Silesia, Katowice, Poland; 4 1st Department and Clinic of Paediatrics, Allergology and Cardiology, Wroclaw Medical University, Wrocław, Poland; The University of Texas Rio Grande Valley, UNITED STATES

## Abstract

The long-range correlations within the current signal time series of the Beta vulgaris vacuolar membrane under the influence of organolead compound (Met_3_PbCl) are investigated. The current time series is transformed into a dwell time series. Then the rescaled range and detrended fluctuations analyses are used. It is shown that the presence of Met_3_PbCl in the solution decreases the mean value of the Hurst exponent and therefore influences the long-range correlations in ionic channel current. This observation is statistically significant. An ion channel model is built and the experimental results reconstructed and analysed.

## Introduction

This paper is the continuation of the researches on the ionic current influenced by organolead compounds [1]. The previous analysis was focused on the changes of the ion current distribution function related to the Met_3_PbCl presence while in the present study the correlations in the dwell-time series of the ion channels are investigated. The main aim of the research is to better understand the influence of organolead compounds on the living organisms. It is important to stress that lead is mainly toxic to living organisms, which was reported in various publications e.g. [2–11]. Lead was reported as being the second most hazardous substance, after arsenic, based on the frequency of occurrence, toxicity, and the potential for human exposure by the Agency for Toxic Substances and Disease Registry [12]. Both acute and chronic lead exposure has the potential to cause many serious systematic effects in people including hypertension, anaemia, immune imbalances, infertility, cognitive deficits, adverse effects on nervous and renal systems, delayed skeletal and deciduous dental development, vitamin D deficiency, and gastrointestinal effects [13]. The accepted consensus is that any level of lead is unhealthy [14]. In developing countries, pregnant women and children are still commonly experiencing today the lead concentration in blood in the range 100–500 *μ*g/L when values <50*μ*g/L are desirable [15]. An important source of lead for humans are plants as the food ingredient [16]. The lead toxicity is reported not only in the human aspects but also in plants. Studies on lead toxicity that have been published in the past decades suggest three main mechanisms of toxicity of Pb^2+^: inhibition of photosynthesis, oxidative stress, and “genotoxicity” including DNA damage and defects in mitosis [17].

Despite the general understanding of the problem and some successes in lead elimination [18] or even development of lead removal methods [19] its industrial usage is, in fact, growing [20], therefore the lead influence on the living organisms is still an important issue. In this research, the vacuolar membrane of *Beta vulgaris* was selected, because the structure and function of this membrane is well-known [21–24] and it can be used as a model membrane. The basic ion currents in vacuolar membranes of higher plants are conducted by the nonselective slow activated cationic channels (SV), fast activated channels (FV), high-selective vacuolar potassium channels. FV channels, in contrast to SV channels, are active at physiological concentration of cytoplasmic Ca^2+^ and blocked by calcium ions, while increasing their concentration in cytoplasm (above 1 *μ*M) [24–26]. It was found that FV channels are also sensitive to the redox potential [27] and other physiological factors [28]. The sensitivity of SV channels to Ca^2+^ ion concentration is related to their involvement in Ca^2+^ homeostasis of the cells [29]. In the conditions most commonly used in researches carried out in patch-clamp technique (symmetrical K^+^ ion concentration and millimolar lumen concentration of Ca^2+^) SV channels are activated at positive membrane potentials. It is worth noticing that in vitro (in patch-clamp experiments) SV channels are activated by the positive (nonphysiological) membrane voltages, whereas in vivo these channels are, in theory, completely deactivated. In the experiments the trimethyllead chloride (Met_3_PbCl) was used since its influence was investigated by standard patch-clamp analysis methods [30]. Moreover, it is soluble in water, so it can be accumulated in the root tissue through the soil environment. In this paper, a new aspect of lead toxicity is verified therefore relatively high lead compound concentration was used.

In the experimental part of the study, the patch-clamp technique was used [31] and the long term correlations of the open—close state time series investigated by the rescaled range analysis (R/S) and detrended fluctuation analysis (DFA). The R/S analysis was proposed by Hurst [32] to analyse changes of river levels but the method appeared to be very useful in the various researches on time series among which were ion channel time series recordings [33, 34]. The main outcome of the R/S analysis is the Hurst exponent, which describes correlation properties of the signal. DFA introduced by [35] is an alternative method to measure Hurst exponent, which was successfully applied to ionic channel analysis [36–39]. In the case of infinite time series, both techniques are expected to give the same results, however, in finite cases the question which one gives a better estimation of the Hurst exponent is still a controversy [40, 41].

## Materials and methods

### The experiment

Electrophysiological measurements presented in the paper were carried out on patches of red beet (*Beta vulgaris* L.) tonoplast. The plants used for the study came from organic farming. Vacuoles were isolated from taproots according to the method described by Coyaud et al [21]. Fresh root was cut with a sharp tool and then washed with the incubation solution, enabling the vacuoles to be directly extruded into the recording chamber (1 ml in volume).

The control bath solution was: 100 mM KCl, 2 mM MgCl_2_, 0.1 mM CaCl_2_, 5 mM MES, 5 mM TRIS and 400 mM sorbitol; pH 7.5 (adjusted by 0.1 N NaOH), osmolarity 656 mOsm. Pipettes were filled with a solution of composition: 100 mM KCl, 2 mM MgCl_2_, 1 mM CaCl_2_, 5 mM MES, 5 mM TRIS, 340 mM sorbitol, pH 5.5, 587 mOsm. The osmolarity of all the solutions used was adjusted under the control of cryoscopic osmometer Semi-Micro_Osmometer K-7400, (Knauer, Germany).

Electrophysiological measurements were carried out using EPC-7 plus amplifier equipped with data acquisition hardware, Instru TECH LIH 8+8 (List-Medical-Electronic, Darmstadt, Germany) and software Patch-Master (HEKA Electronic, Lambrecht, Germany). Signal was probed every 50 *μ*s and analog filtered by Bessel filter (in the device) at cut-off frequency 1 kHz. Transmembrane voltage was controlled in voltage clamp condition. Membrane polarity was consistent with Bertl et al. convention [42].

Micropipettes were made from borosilicate glass tubes (Kimax-51, Kimble Products, Toledo, Ohio, USA) using two stage puller (Model L/M-3-PA, List medical, Germany), fire polished with a microforge CPZ 101 (List medical, Germany) and coated with Sylgard (Dow Corning, Midland, MI, USA). The resistance of micropipette filled with a pipette solution was in the range of about 2–4 MΩ, and seal resistance 4–20 GΩ.

Microscopic currents were recorded in the inside-out configuration (cytosolic side out patch), which was obtained in the following way. First starting from the vacuole-attached configuration and using electroporation of membrane within a micropipette (applying short-time voltage pulse in the range of 300 to 900 mV), a whole vacuole system was obtained. Next, after detachment of the patch from tonoplast with a quick micropipette movement, we got the inside-out configuration.

Measurements protocol was as follows. The voltage was maintained as a constant function in a given time interval. Starting from zero (10 s) the potential was subsequently changed to -50 mV (operating voltage) and kept on this value for 10 seconds, after which it was switched back to zero for another 10 s. Such cycle was repeated with the gradation of operating voltage every 10 or 20 mV in the range (- 50 mV, 100 mV) and in some experiments only 80 and 100 mV. Effect of 100 *μ*M trimethyllead (Met_3_PbCl) on SV channels activity were studied. Due to the duration of our experiments (from a few to several mins) we decided to use higher lead concentration than those present in nature). During experiments, the control solution was changed for a new one of the same composition, supplemented by trimethyllead.

After doing several such series of records in the control the same vacuole was incubated in a solution containing trimethyllead. The exchange of incubated media was carried out by continuous perfusion of the measuring chamber using an infusion pump (SP 200, World Precision Instruments, USA). Replacement time of the incubation solution was approx. one minute.

Experiments were carried out in the room temperature (22±1°C). For the data analysis, the ion current recordings were pre-normalized by subtracting the leak currents (the zero offset).

### Data analysis

Data from 28 different vacuolar patches were analyzed. On most of them due to the instability of the patch-micropipette layout for a longer period (over ca. 10 minutes), the data comes from individual protocols (control and then solution supplemented with Met_3_PbCl). If the patch was sufficiently stable, the measurement protocol was repeated several times in control and then in the presence of a modifier. The analysis included a total of 80 individual traces (each of them with a duration of 10 seconds), 40 for the control and 40 for the modifier, respectively. The activity of SV channels in the membrane was usually determined by recording the macroscopic current (in the whole vacuole system, before forming the inside out). This current shows characteristic typical for SV channels: sigmoidal time course with activation constant of seconds and rectifying current-voltage characteristics (data not shown). Recorded microscopic currents were consistent with what is observed in the whole system and in good agreement with the data of other authors—conductivity of single-channel—72 pS and voltage-dependent gating (activity were observed at a voltage above 40 mV).

In the experiment, the current through an ion channel was measured for the following external potential: (-50 mV, 100 mV), with step 10 mV. However, the best results with clearly open and closed states were observed for the external potential equal 80 mV and these time series were analysed within this paper. The experiment was repeated 40 times (standard bath solution) and 40 times in the bath solution with 0.1 mM of Met_3_PbCl.

Each analysed time series consists of 2 ⋅ 10^5^ data points with the resolution of 0.05 ms (10 s recordings). The time series registered during the patch-clamp measurements were verified against the presence of more than one active channel and stationarity of the ionic channel recordings.

The problem of sometimes observed instability of the functioning of the channel, in particular, spontaneous loss of channel activity over time called run-down is not sufficiently recognized in the literature so far. It should be emphasized that this phenomenon is rather not the result of the destruction of the channel structure because the current through a single channel does not change, while its activity is temporarily lost. Furthermore, the latter is also correlated with the loss of macroscopic current.

A large number of ion channels, in particular potassium channels, are regulated by substances contained in the cytosol, such as nucleotides, calcium ions and other signaling molecules. In patch clamp experiments, homeostasis of membrane contact with the intracellular environment is disturbed by washing out these substances as a result of membrane perforation (whole-cell) or patch detachment.

In general, the molecular mechanism responsible for the run down has not been well understood, however, this phenomenon has been thoroughly studied on L-type calcium channels. A binding site (C-terminal sequence 1572–1651 of the *α*_1*C*_ subunit for the intracellular modulator, calpastatin, responsible for the run down effect was found in this channels [43].

In the case of SV channels of higher plants [44, 45] observed run-down of ion current was rather low on the level of 18%–19% within several minutes of observation. Much higher influences were observed in animal cells [46].

Some solution to the run down problem in patch clamp experiments (in whole cell configuration) seems to be the use of pore-forming substances, such as antibiotics (amphotericin B, nystatin). The pores thus created allow electrical contact between the micropipette solution and the interior of the cell without disturbing its homeostasis, due to the possibility of only small ions penetrating the pore formed [47]. Another method of the run-down prevention is the usage of dedicated compounds e.g. by adding DTT [48], ATP [49] or DTP [50], however none of the authors does not discuss the recovery of statistical properties of the ion channels. Therefore, the protocol, which allowed to analyse ion current without additional components was chosen.

**Fig 1:**
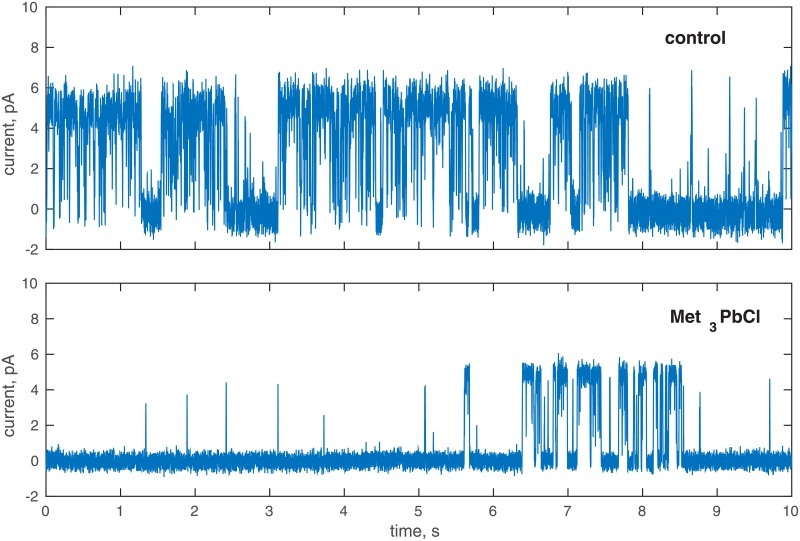
An example of the registered ionic current in the control solution and in the presence of Met_3_PbCl. Membrane potential was 80 mV.

The probability distribution functions (see Sec. *Probability density of ionic current*) of the registered signals were obtained and the cases with two maxima chosen i.e. with one active ionic channel. The stationarity of the time series was verified by the bootstrap method [51] and the time series with fluctuations of the 90 and 10 percentile exceeding 5% were rejected. After the inspection of the data, the analysis was continued on the 35 recordings at the standard bath solution and 23 time series at the solution with 0.1 mM of Met_3_PbCl. The examples of the registered time series are presented in Fig 1. For the chosen time series the value of the threshold was obtained and the current time series were transformed into the dwell-time series. Finally, the long-range correlations were investigated by R/S and DFA analyses.

### Probability density of ionic current

**Fig 2:**
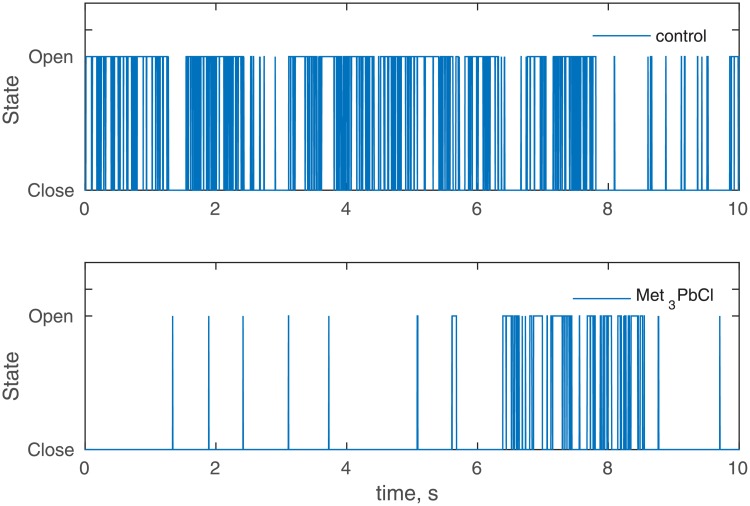
PDF’s of the time series presented in Fig 1 fitted by the kernel distribution function. Membrane potential was 80 mV.

**Fig 3:**
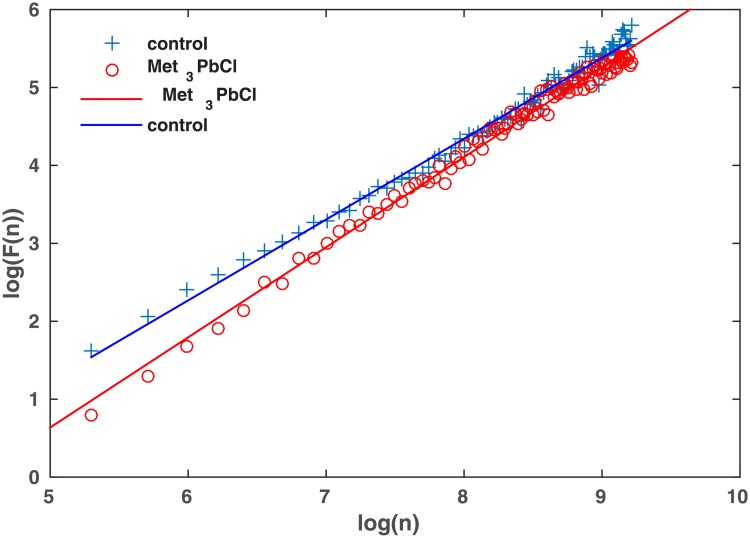
The dichotomous open-close state signal obtained from the signal presented in Fig 1. Membrane potential was 80 mV.

A histogram is the simplest probability functions approximation, however, the results are rather rough. In order to resolve this ambiguity the kernel density estimator with the Gaussian kernel density was used [52, 53]. The examples of the obtained probability distribution functions (PDF) are presented in Fig 2. The procedure of the threshold current approximation proposed by [36, 37] was modified due to the number of time series analysed (more than a thousand samples). The threshold current between the open and close states was found as a local minimum of the fitted PDF. Finally, the ionic current signal was converted into the dichotomous signal (open and close states) and the time series of open and close states times were obtained. The example of the converted time series is presented in Fig 3.

### R/S analysis

The detailed description of rescaled range analysis (R/S) can be found in various books on the fractal analysis e.g. [54]. Here the key elements are presented. Let denote the time series by A and its elements as {*a*_1_, *a*_2_, …, *a*_*n*_}, then

1. Create the mean adjusted time series *b*_*i*_ = *a*_*i*_ − 〈*A*〉, where 〈*A*〉 is the mean value of the time series.
2. Calculate cumulative time series: $c_{t}=\sum_{i=1}^{t}b_{i},\;t=1,2,3\ldots ,n$.
3. Create the range series: *r*_*t*_ = max{*c*_1_, *c*2, …, *c*_*t*_} − min{*c*_1_, *c*2, …, *c*_*t*_}, *t* = 1, 2, 3…, *n*.
4. Create the standard deviation time series: $s_{t}=\sqrt{\frac{1}{t}\sum_{i=1}^{t}\left(a_{i}-u\right)}$, where *t* = 1, 2, 3…, and $u=\frac{1}{t}\sum_{i=1}^{t}a_{i}$.
5. Calculate R/S time series: ${\left(\frac{R}{S}\right)}_{t}=\frac{r_{t}}{s_{t}}$.
6. Finally the power law ${\left(\frac{R}{S}\right)}_{t}∼t^{H}$ is fitted to the transformed time series and the Hurst exponent (H) estimated.

In the case of the persistent time series $H>\frac{1}{2}$, while for the antipersistent time series $H<\frac{1}{2}$.

### DFA analysis

The detrended fluctuation analysis (DFA) is an alternative method for Hurst exponent estimation. Details can be found in e.g. [55, 56].

1. The procedure starts form the transformation of a time series A (of *N* samples), {*a*_1_, *a*_2_, …, *a*_*N*_} into a cumulated mean adjusted time series: $X_{t}=\sum_{i=1}^{t}\left(a_{i}-\langle a_{i}\rangle \right)$, then
2. the time series is divided into *n* equal size boxes, and
3. in every box a local trend *y*_*n*_ is fitted. In fact any function could be used to approximate the trend, but in practice the linear function is the most popular choice. The higher order polynomials are rarely used e.g. [57].
4. The trend is subtracted from the integrated signal and its fluctuation calculated: $F\left(n\right)=\sqrt{\frac{1}{N}\sum_{k=1}^{N}{\left[X_{k}-y_{n}\left(k\right)\right]}^{2}}$. *F*(*n*) is calculated for all possible sizes of boxes.
5. Finally the power law function *F*(*n*)∼*n*^*α*^ is fitted. (Usually by fitting linear function to the log-log transformed plot.)

The interpretation of the results is: (i) if *α* < 0.5, then the anti-correlated time series is observed, (ii) *α* ≃ 0.5 the uncorrelated white noise is measured, (iii) *α* > 0.5 the correlated time series is observed.

The main difference between R/S and DFA is that R/S removes a constant trend from the data while DFA subtracts the linear trend.

## Results

**Fig 4:**
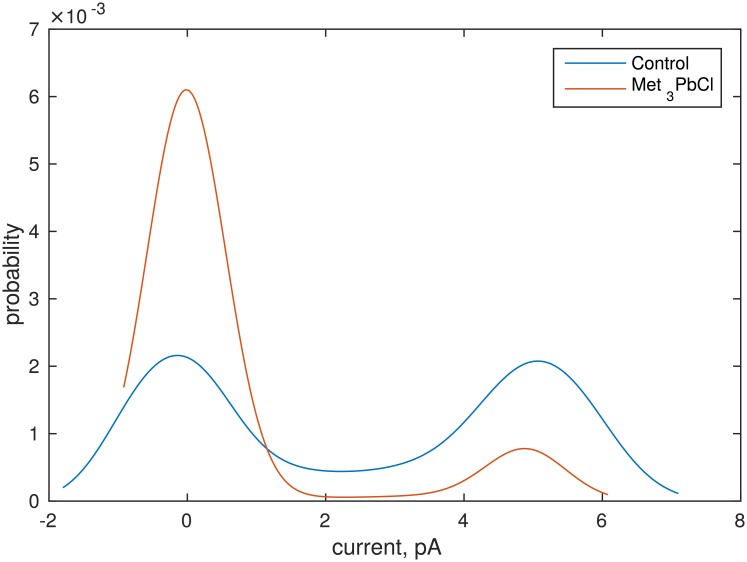
The DFA of the signal presented in Fig 1. *n* denotes the box size, while *F*(*n*) the fluctuations. Membrane potential was 80 mV.

One example of DFA analysis is presented in Fig 4. The results of the DFA and R/S analysis are presented in Tables 1 and 2. The Hurst exponents for both methods applied and in all considered experimental data significantly exceed the value 0.5 showing the existence of long-range correlations of open-close states. Analysing the results obtained by R/S and DFA it is observed that the Hurst exponents calculated by R/S analysis are smaller than those received by DFA. The difference between mean values of the Hurst exponent at R/S analysis is equal 0.07 while for DFA analysis 0.13. However, the most important result is that the observed changes in long-correlation being the result of the Met_3_PbCl presence are statistically significant. The one way ANOVA analysis have been applied and the p-value of the null hypothesis stating that there is no difference of the Hurst exponent of the ion current in the control solution and in the presence of Met_3_PbCl is equal 1.2 ⋅ 10^−5^ at R/S analysis and even 6.4 ⋅ 10^−13^ at DFA analysis. Therefore, the null hypothesis is rejected and the alternative accepted. Due to the uni-vocal result, there comes the question on the possible mechanism of the *Met*_3_*PbCl* influence. This problem is analysed in the following section.

**Table 1:** The mean value and the standard deviation of the Hurst exponent approximation obtained by R/S analysis. Additionally the statistical significance of the difference between means is presented. Membrane potential was 80mV.

	control	Met_3_PbCl
mean	0.85	0.78
std	0.03	0.03
ANOVA p-value		1.2 ⋅ 10^−5^

**Table 2:** The mean value and the standard deviation of the Hurst exponent approximation obtained by DFA analysis. Additionally the statistical significance of the difference between means is presented. Membrane potential was 80 mV.

	control	Met_3_PbCl
mean	1.05	0.93
std	0.07	0.13
ANOVA p-value		6.4 ⋅ 10^−13^

## Channel model

Within the present work, the long memory effects are investigated therefore the problem of developing a model is interesting itself and the reader may refer to [58]. Within the present analysis, the model developed in [58] is used. For the convenience of the reader, the key model features and the algorithm generating the dwell time series are presented here. The main advantage of the model is that it does not only reconstruct the desired open-close state probability but also generates appropriate autocorrelations among the states. The model is defined on a 1-dim lattice with given boundaries and the arbitrarily chosen point dividing the lattice space into open and close states. The open-close states time series are generated by random walk on a lattice with an external potential and a threshold between open and close states spaces.

The conformational space is defined on 1-dim lattice with N-nodes. The ends of the lattice are denoted as *B*_*MIN*_ and *B*_*MAX*_. The reaction coordinate (RC) corresponding to the state of the channel gate is placed on the lattice and at each step of the simulation RC can be moved by one node. The range of the accessible nodes is limited by movable boundaries *B*1 and *B*2. The motion of these boundaries corresponds to the thermal fluctuations in the membrane thickness and internal strains within protein segments influencing the conformational space of open and close states. They are synchronised in directions (i.e. if the spaces corresponding to the open states shrinks the same happens to the close states space). At the centre of the lattice the threshold point (TP) is placed. This point divides the conformational space into open and close spaces. During the simulations, there are two movable elements: RC, B1 and B2 (B1 and B2 are synchronised). Since there is a significant difference in mass of the fluctuating membrane and the mass of the putative activation gate the fluctuation rate of both parameters are on different time scales. The change of the boundaries is performed after a given number of iterations according to the formula:

$$\begin{array}{c} D_{B}=\frac{D_{RC}}{600}, \end{array}$$ (1)

which was estimated on the experimental data (*D*_*B*_ is the boundary diffusion coefficient while *D*_*RC*_ denotes reaction coordinate diffusion coefficient) [58]. The RC perform on the lattice a biased random walk. The probabilities of decreasing (q) or increasing (p) position of RC are given by the Eqs (2) and (3) [59].

$$\begin{array}{c} q=\frac{1}{2}+\frac{\Delta U}{4kT} \end{array}$$ (2)

$$\begin{array}{c} p=\frac{1}{2}-\frac{\Delta U}{4kT} \end{array}$$ (3)

Where *k* is the Boltzmann constant, *T* the absolute temperature and Δ*U* a potential energy difference within a lattice step centred around RC. In order to recover appropriate drift force (being the result of the external potential on the membrane e.g. *V* = 80*mV*) the potential *U*(*x*) is postulated in the following form [58]:

$$\begin{array}{c} \left\{ \begin{array}{c} U\left(x\right)=\left(x-B1\right)\cdot A+U_{B1};x\in \left\langle B1;TP-1.5\right)\\ U\left(x\right)=\left(x-\left(TP-1.5\right)\right)\cdot B+U_{TP-1.5};x\in \left\langle TP-1.5;TP\right)\\ U\left(x\right)=\left(x-TP\right)\cdot \left(-B\right)+U_{TP};x\in \left\langle TP;TP+1.5\right\rangle \\ U\left(x\right)=\left(x-\left(TP+1.5\right)\right)\cdot A+U_{TP-1.5};x\in \left\langle TP+1.5;B2\right)\\ A=\frac{U_{TP-1.5}-U_{B1}}{TP-1.5-B1}\\ B=\frac{U_{TP}-U_{TP-1.5}}{TP-(TP-1.5)} \end{array},\right. \end{array}$$ (4)

where *B*1 and *B*2 are the locations of the left and right boundaries, respectively, *TP* is the threshold point position, *U*_*B*1_, *U*_*TP*_ and *U*_*TP*−1.5_ are values of the potential at the given points. It is also assumed that the potential changes its value linearly and *A* and *B* are the slope coefficients defining potential slopes on the intervals (*B*1;*TP* − 1.5) and (*TP* − 1.5;*TP*), respectively. After each iteration step the position of RC is checked and if it is on the left of TP then open state is recorded in generated time series otherwise the close state is registered.

The lattice size was set to 2*B*_*MAX*_ + 1 = 29 nodes—determining the maximum size of the open and closed state spaces. The starting position was set at the closed neighbourhood of the threshold TR (*RC* = {−1, 0, 1}). In fact, the choice of the initial position does not influence the results of simulations. Assuming that the iteration step of the simulation necessary to move RC is equal to 1 then the boundaries positions *B*1 and *B*2 were changed every 600 iteration steps to satisfy Eq (1).

While performing the simulations the experimental limitations were taken into account: the ion channel time series were of limited size (i.e. 2 ⋅ 10^5^ data points), the statistical parameters of ion channel time series (i.e. open probability, and Hurst exponent obtained by R/S and DFA analyses) have a dispersion—due to the biological variability and time series size. Moreover, the theoretical model is in fact a non-linear model with four parameters. Therefore, the possibility that there are more than one solution (a parameter set under which the simulations generate time series of required properties) should not be excluded. In order to find multiple solutions, instead of optimization technique [58] the scanning of the state space was performed. The initial parameter combination were {*U*_*B*1_ = 1*kT*, *U*_*B*2_ = 1*kT*, *U*_*TP*_ = 1*kT*, *U*_*TP*−1.5_ = 1*kT*} till {*U*_*B*1_ = 25.5*kT*, *U*_*B*2_ = 25.5*kT*, *U*_*TP*_ = 25.5*kT*, *U*_*TP*−1.5_ = 25.5*kT*} with resolution 0.5*kT*, so 6.25 ⋅ 10^6^ states where examined. The temperature was set *T* = 300*K*. The others parameters agreed with [58] (*B*_*MAX*_ = −*B*_*MIN*_ = 14, *TP* = 0).

Since the Hurst exponent analysis (both R/S and DFA) are computationally complex the simulations were divided into two steps. During the first scan the opening probabilities were calculated and after filtering the results such that the opening probabilities were in the interval *average opening probability ± standard deviation*, then the simulations were repeated for the filtered parameter set. At this stage for each set of parameters twelve samples of time series were generated and the DFA and R/S analyses were performed. Finally the parameters sets matching the results obtained on the experimental data (Tables 1 and 2) were collected. The simulation parameters that generates time series with expected open probability and Hurst exponent are presented in Tables 3 and 4.

**Table 3:** The parameters (in kT units) of the theoretical model reconstructing time series of the properties of the ion channel in the presence of Met_3_PbCl.

parameters (UB1; UB2; UTP; UTP15)	open prob. *p*±*σ*	H by R/S *h*±*σ*	H by DFA *h*±*σ*
(1; 1; 24.5; 24)	0.019±0.005	0.78±0.02	1.19±0.03
(1; 1.5; 25.5; 25.5)	0.018±0.004	0.77±0.02	1.22±0.03
(1; 10.5; 24.5; 24)	0.020±0.006	0.78±0.02	1.19±0.03
(1; 12.5; 24; 22.5)	0.024±0.006	0.78±0.02	1.13±0.03
(1; 14; 24.5; 25.5)	0.017±0.004	0.77±0.03	1.24±0.13
(1; 16; 23; 23)	0.019±0.004	0.77±0.03	1.22±0.03
(1; 17.5; 24.5; 24.5)	0.018±0.005	0.77±0.02	1.21±0.03
(1; 22; 25.5; 25)	0.021±0.007	0.77±0.02	1.19±0.04
(1; 24; 25; 24.5)	0.021±0.004	0.77±0.02	1.19±0.03
(1; 24; 25.5; 24)	0.021±0.007	0.78±0.02	1.14±0.03
(1.5; 5; 23.5; 23)	0.019±0.006	0.77±0.02	1.19±0.03
(1.5; 5; 25.5; 25.5)	0.018±0.005	0.78±0.02	1.20±0.03
(1.5; 11; 22.5; 22.5)	0.018±0.005	0.77±0.02	1.22±0.03
(1.5; 8.5; 25; 24)	0.021±0.006	0.78±0.02	1.17±0.05
(1.5; 9.5; 21.5; 23)	0.016±0.005	0.77±0.02	1.25±0.05
(1.5; 13.5; 25; 23.5)	0.023±0.005	0.78±0.02	1.15±0.04
(1.5; 14.5; 21.5; 22)	0.017±0.004	0.77±0.02	1.23±0.03
(1.5; 20.5; 22; 23)	0.017±0.005	0.77±0.02	1.24±0.03
(1.5 15 23.5 24)	0.018±0.006	0.77±0.02	1.23±0.03
(1.5; 15; 25; 24.5)	0.020±0.004	0.77±0.02	1.20±0.03
(2; 7.5; 24; 23.5)	0.018±0.006	0.77±0.02	1.19±0.03
(2; 9; 24; 23)	0.022±0.007	0.78±0.02	1.17±0.03
(2; 13; 24.5; 22.5)	0.023±0.006	0.78±0.02	1.11±0.04
(2; 15; 20.5; 19)	0.019±0.006	0.77±0.02	1.14±0.04
(2; 16; 23.5; 23.5)	0.021±0.007	0.77±0.02	1.22±0.03
(2; 23; 21.5; 20.5)	0.020±0.007	0.77±0.02	1.17±0.03
(2; 24; 21.5; 21)	0.021±0.005	0.77±0.02	1.19±0.02
(2.5; 19.5; 24; 22.5)	0.025±0.007	0.78±0.02	1.14±0.03
(2.5; 21; 24.5; 25.5)	0.017±0.004	0.77±0.02	1.25±0.03
(2.5; 24; 24; 24)	0.018±0.006	0.78±0.02	1.2±0.03
(3; 5; 24; 23)	0.020±0.006	0.77±0.02	1.16±0.04
(3; 6.5; 24; 24.5)	0.016±0.005	0.77±0.02	1.23±0.03
(3; 11; 22; 22.5)	0.018±0.005	0.77±0.02	1.22±0.03
(3; 12.5; 24; 23)	0.021±0.006	0.78±0.02	1.16±0.04
(3; 14; 22.5; 22)	0.018±0.006	0.77±0.02	1.19±0.04
(3; 15.5; 24.5; 23.5)	0.020±0.007	0.77±0.02	1.17±0.03
(3; 17; 24; 23.5)	0.019±0.006	0.77±0.02	1.20±0.03
(3; 17.5; 25.5; 25.5)	0.020±0.006	0.77±0.02	1.21±0.03
(3.5; 17.5; 24.5; 24.5)	0.018±0.005	0.77±0.02	1.22±0.03
(4.5; 25; 24; 22.5)	0.021±0.007	0.78±0.02	1.14±0.03
(5; 5.5; 25.5; 25)	0.017±0.004	0.77±0.02	1.19±0.03
(5; 25; 20.5; 20.5)	0.017±0.004	0.78±0.02	1.21±0.03
(6.5; 1.5; 25.5; 25)	0.020±0.005	0.77±0.02	1.19±0.02
(8.5; 13; 24.5; 24.5)	0.020±0.006	0.78±0.02	1.21±0.03

**Table 4:** The parameters (in kT units) of the theoretical model reconstructing time series of the properties of the ion channel in the control solution.

parameters (UB1; UB2; UTP; UTP15)	open prob. *p*±*σ*	H by R/S *h*±*σ*	H by DFA *h*±*σ*
(4.5; 23.5; 1; 3)	0.447±0.038	0.80±0.03	0.77±0.03
(10.5 24 7.5 9.5)	0.413±0.023	0.79±0.02	0.77±0.03
(21.5; 11.5; 16; 18.5)	0.485±0.033	0.82±0.03	0.85±0.04
(23.5; 5; 19; 21.5)	0.409±0.029	0.80±0.02	0.80±0.02
(25.5; 23; 20; 22.5)	0.489±0.046	0.81±0.03	0.84±0.03

The simulations parameters of the numerical model generating dwell time series (Table 3) of the properties similar to the ionic current time series at the presence of Met_3_PbCl are characterised by low values of the considered set of UB1 potential and are at the range from 1 kT up to 8.5kT. The potential threshold at TP takes high values at the interval from 19.5kT up to 25.5kT. Similar values are observed for UTP15, which is responsible for the potential just before and after TP, and the values are in the range 20.5kT to 25.5kT. The difference between UTP and UTP15 is relatively small considering the range of the simulation parameters and vary from -1kT up to 2kT, but most of the collected simulation parameters are characterised by the positive difference between UTP and UTP15. The significant spread between UB1 and UTP15, which in fact “keeps” RC at the close state side of the lattice. It is also noticeable that within the chosen range of simulation parameters there are more parameters sets which reconstruct the behaviour of the ionic channel at the presence of Met_3_PbCl than in control solution. Another interesting observation is the difference between the Hurst exponent obtained by R/S and DFA methods. In Table 3 the Hurst exponent obtained by R/S is smaller than received by DFA. The similar difference is observed for experimentally observed time series. Although, both methods should give similar results it is true only in an infinite time series [40]. In the case of real observations, there is a “sample size effect” and both methods give different results. However, the difference between Hurst exponent obtained by both methods is analogous in experimental and simulated time series.

The parameters of the model reconstructing the time series of the ion channel in the control solution are presented in Table 4. In this case, the UB1 potential takes much higher values, which lies in the interval 4.5kT to 25.5kT however the high values (>20kT) prevail. Particularly interesting is the analysis of the threshold potentials UTP and UTP15. These potentials do not define a threshold but rather a potential well, which forms a “trap” for RC with the depth in the range 2kT and 2.5kT. In three out of five sets have potential UB2>UB1.

## Discussion

The transport mechanisms, within ion current are crucial to cell biology. The recent researches indicate long-range correlations in the open-close state time series of the single ion channel currents [36–39]. The main aim of the present study is to verify whether the reported long-range correlations are sensitive to the presence of organolead. In the analysis, the ionic time series were registered by the patch-clamp technique and subsequently transformed into the dichotomous signal, which was finally converted into the dwell time series. The transformed series were investigated by R/S and DFA analyses. Besides the result that the open-close state time series of SV channels of the vacuolar membrane is persistent (*H* > 0.5), which was also reported in similar studies [39, 60] it has been shown that the long-range correlations are sensitive to the presence of Met_3_PbCl. This result is new and provides two important observations: firstly the long correlations in ion channels currents might be sensitive to various compounds and more specific is that the lead influences such correlations. The origins of the long-range correlations are still a subject of investigations. The observed phenomenon was further investigated by the numerical model reconstructing dwell time series of required properties. The sets of model parameters generating the time series of the characteristic of the ion channel current at the control solution and at the presence of Met_3_PbCl were found. The most important difference of the sets was that those reconstructing the organolead compound influence were characterised by significantly lower values of UB1 potential such that instead of potential well the threshold is observed.

It should be stressed that the disturbance of the long-term correlations could be considered as a destabilization element, which in consequence may results in an increase of vulnerability of the organisms.

Besides the direct result of our study i.e. the observation that Met_3_PbCl change long range correlation in ion channel current we would like to draw attention on another problem. In physical systems the existing correlations are the results of interactions. So, if the long memory of ion channels has biological significance than there exists a mechanism responsible for it. This hypothesis is very intriguing because on the microscopy level there are not many reasons that the molecules might “remember” its previous state. On the other hand, this study shows that some compounds indeed change long memory parameters, so the ion channel models should include mechanisms properly reconstructing also this aspect.

The obtained results open a new area of research showing that long correlations of ion channels may be influenced by various compounds and this aspect should be also a subject of investigations.
